# Apoptin induces pyroptosis of colorectal cancer cells via the GSDME-dependent pathway

**DOI:** 10.7150/ijbs.64350

**Published:** 2022-01-01

**Authors:** Zirui Liu, Yiquan Li, Yilong Zhu, Nan Li, Wenjie Li, Chao Shang, Gaojie Song, Shanzhi Li, Jianan Cong, Tingyu Li, Zhiru Xiu, Jing Lu, Chenchen Ge, Xia Yang, Yaru Li, Lili Sun, Xiao Li, Ningyi Jin

**Affiliations:** 1 College of Veterinary Medicine, Jilin University, Changchun, 130062, China.; 2 Changchun Veterinary Research Institute, Chinese Academy of Agricultural Sciences, 130122, China.; 3 Academician Workstation of Jilin Province, Changchun University of Chinese Medicine, Changchun, 130021, China.; 4 Jiangsu Co-innovation Center for Prevention and Control of Important Animal Infectious Diseases and Zoonoses, Yangzhou, 225009, China.; 5 Department of Head and Neck Surgery, Tumor Hospital of Jilin Province, Changchun, 130012, China.

## Abstract

Apoptin is a small molecular weight protein encoded by the VP3 gene of chicken anemia virus (CAV). It can induce apoptosis of tumor cells and play anti-tumorigenic functions. In this study, we identified a time-dependent inhibitory role of apoptin on the viability of HCT116 cells. We also demonstrated that apoptin induces pyroptosis through cleaved caspase 3, and with a concomitant cleavage of gasdermin E (GSDME) rather than GSDMD. GSDME knockdown switched the apoptin-induced cell death from pyroptosis to apoptosis *in vitro*. Furthermore, we demonstrated that the effect of apoptin on GSDME-dependent pyroptosis could be mitigated by caspase-3 and caspase-9 siRNA knockdown. Additionally, apoptin enhanced the intracellular reactive oxygen species (ROS), causing aggregation of the mitochondrial membrane protein Tom20. Moreover, bax and cytochrome c were released to the activating caspase-9, eventually triggering pyroptosis. Therefore, GSDME mediates the apoptin-induced pyroptosis through the mitochondrial apoptotic pathway. Finally, using nude mice xenografted with HCT116 cells, we found that apoptin induces pyroptosis and significantly inhibits tumor growth. Based on this mechanism, apoptin may provide a new strategy for colorectal cancer therapy.

## Introduction

Oncolytic Adenoviruses are novel and highly effective gene delivery vectors which can be modified to express specific genes in the target cancer cell, eventually causing cell lysis and death 1.Our laboratory developed an Ad-vp3 (Ad5-apoptin) that expresses apoptin. Another Ad-vt (Ad5-apoptin-hTERTp-E1a) that can continuously express apoptin in the cancer cell via an hTERT promoter-driven was also developed. Apoptin is a proline rich VP3 structural protein of chicken anemia virus (CAV). Once expressed in cancer cells, it will induce mitochondria to release cytochrome c, thus inducing activated caspase-3 and leading to apoptosis 2, 3. It has been found to induce apoptosis in a variety of tumor cell lines regardless of p53 regulation, but not in healthy normal cells 4. Apoptin expression in cancer cells using Oncolytic Adenoviruses might provide a tumor-specific method in clinical cancer therapy.

Apoptosis is usually mediated by intracellular cysteine proteases (caspases), which initiate and execute the apoptosis process 5. The expression of apoptin has been shown to lead to the activation of caspases 6. Apoptin-mediated cell death is independent on the death receptor because cells lack FDD or caspase-8, which is a key regulator of exogenous apoptotic pathways 7. Moreover, Apoptin-mediated apoptosis is strongly influenced by the mitochondrial pathway regulators, such as Apaf-1, which has a strong protective effect on tumor cells. In addition, apoptin triggers the release of cytochrome c and the activation of the caspase-9 8. Due to its tumor-specific toxicity, apoptin may require additional interaction partners to activate specific signaling pathways in cancer cells. In fact, many molecules interact with apoptin and seem to be important for the nuclear localization of apoptin or its tumor-specific cytotoxicity 9, however, the mechanism of cell death induction is still unclear.

Apoptosis, necrosis, and autophagy have been shown to be related to apoptin induced cell death. Apoptin induced cell death is caused by the mitochondrial apoptosis pathway and the ROS pathway 10, 11. In this study, we confirmed that apoptin triggers another type of programmed cell death (PCD), named pyroptosis, via the ROS pathway. When GSDMD (Gasdermin D) is cleaved by activated caspase-1 or caspase-4/5/11, and an N-terminal pore-forming domain (PFD) is formed and non-specific holes are drilled on the surface of the cell membrane, resulting in membrane rupture and the release of activated cytoplasmic IL-1β andIL-18 12-15. However, a growing number of studies have found that GSDMD is not the only executor of pyroptosis, GSDME (a member of gasdermins family, also known as DFNA5) can also form membrane oligomeric pores on the cell membrane after being cleaved by activated caspase-3 16, 17. Although pyroptosis's anti-infective effects on immune cells lead to a low-level or high-level inflammatory response, it is unclear whether its induction can be used for cancer treatment.

Colorectal cancer (CRC) is the second most deadly cancer and third most malignant tumor in the world. In 2018, there were 1.8 million new cases of CRC, and 881,000 reported deaths, accounting for nearly 10% of new cancer cases and deaths worldwide 18. Meanwhile, the 5-year survival rate for colorectal cancer is 64%, while the 5-year survival rate for metastatic colorectal cancer is 12% 19. Typically, the ideal treatment for CRC is a complete resection of the tumor and metastases, most of which requires surgical intervention 20. Nearly 25% of CRC cases are diagnosed as advanced stages with metastases, and about 20% of cases may develop metastases that are inoperable with poor prognosis 21-24. For this situation, radiotherapy and chemotherapy to minimize and stabilize tumors are the main therapeutic strategies for these patients 25, 26. However, chemotherapy has certain limitations, such as systemic toxicity, adverse reaction rate, unpredictable innate and acquired drug resistance, low tumor specific, and selectivity. Therefore, it is very important to develop new CRC treatment strategies. Our study reveals a mechanism by which apoptin induces pyroptotic death of Colon cancer cells, and indicates that the recombinant oncolytic adenovirus expressing apoptin has the potential application as a novel treatment for CRC.

## Materials and methods

### Reagents and antibodies

The anti-GAPDH (Cat# 5174), anti-caspase-1 (Cat# 2225), anti-cleaved-caspase-3 (Cat# 9664), anti-cleaved-caspase-9 (Cat# 9505), anti-cleaved-caspase-6 (Cat# 9761), anti-cleaved-caspase-7 (Cat# 8438), anti-cleaved-caspase-8 (Cat# 4790), anti-Bax (Cat# 2774), anti-Cytochrome c (Cat# 4272), anti-PARP (Cat# 9542), anti-tom20 (Cat# 42406), antibodies were purchased from Cell Signaling Technology. Anti-GSDME (Cat# ab215191), and anti-GSDMD (Cat# ab209845) antibodies were purchased from abcam. The anti-rabbit (Cat# 7076) and anti-mouse (Cat# 7074) secondary antibodies were purchased from Cell Signaling Technology. Hoechst 33342 (Cat# 1399), ROS detection CM-H2DCFDA (Cat# C6827), were purchased from ThermoFisher Scientific. The Apoptosis Detection FITC Annexin V was purchased from BD Biosciences. The Caspase-3-specific inhibitor Z-DEVD-FMK (Cat# HY12466), staurosporine (Cat#HY-15141) and GSH (Cat#HY-D0187) were purchased from Master of Small Molecules. Caspase-pan inhibitor Q-VD was purchased from Sigma Aldrich. The CytoTox 96 Non-Radioactive Cytotoxicity Assay (Cat#G1780) was purchased from Promega. The Cell Counting Kit-8 and 4-(4-(Dihexadecylamino)styryl)-N-methylpyridinium iodide were purchased from Dojindo.

### Cell culture, viruses, and animals

The human colon cancer cell lines, HCT116, Sw-620, Sw480, and the lung carcinoma cell lines, 446 and 226, were cultured in RPMI 1640 medium (Gibco). The hepatoma cell lines hepG2, 7721, huh7, and the breast cancer cell lines, MCF-7 and caco2 were cultured in DMEM medium (Gibco). The cells were supplemented with 10% FBS (Gibco) and incubated at 5% CO 2 at 37 °C. All cells were acquired from the Cell Bank of the Shanghai Institute for Biological Sciences (Shanghai, China). The recombinant oncolytic adenoviruses, Ad-mock, Ad-vp3 and Ad-vt, were constructed and preserved in our lab (Laboratory of Molecular Virology and Immunology, Institute of Military Veterinary Medicine, Academy of Military Medical Science, Changchun, China).

### Western blot analysis

HCT116 cells were treated with the recombinant oncolytic adenoviruses, Ad-mock, Ad-vp3, and Ad-vt, at a dose of 100 MOI. At 12, 24, 36, 48, and 72 hours, the cells were collected and centrifuged to remove the supernatants. Then cells were lysed in IP buffer, and 30μg of total protein from each sample was loaded onto SDS-PAGE and transferred onto PVDV membranes. The membrane was sealed with TBST containing 5% skim milk for 2 hours, and the corresponding primary antibody was added overnight at 4°C. Before incubation with the goat anti-rabbit secondary antibody or the goat anti-mouse for 40 minutes. Afterward, the membranes were washed 3 times with TBST for 10 minutes each. Finally, the protein was detected by enhanced chemiluminescence ELC Western Blot substrate.

### Microscopy

To observe the typical characteristic of pyroptotic cells, HCT116 cells were seeded in a 6-well plate (3 × 10^5^). After a 36h treatment with recombinant oncolytic adenoviruses, bright field images of the cells were captured using the EVOS M5000 microscope (Invitrogen). To examine the pyroptotic cell membranes, the cells were stained with PlasMem bright green (Dojindo), and images were captured by laser scanning confocal microscope (ZEISS).

### Apoptosis detected by flow cytometry and tunnel assay

To detect apoptosis in HCT116 cells, flow cytometry was performed. The cells were treated with trypsin, collected, washed thrice with PBS and used for apoptosis detection using the Annexin V-FITC/PI apoptosis detection kit and according to the manufacturer's instructions. The percentages of positive cells were analyzed using a Beckman Coulter flow cytometer. Each experiment was repeated three times. Tunnel (terminal deoxynucleotidyl transferase dUTP nick end-labeling) assay was also performed to detect apoptosis using the TUNEL Apoptosis Assay Kit (Solarbio) to detect positive cells and following to manufacturer's recommendations.

### Intracellular ROS

The intracellular ROS levels were detected by ROS detection CM-H2DCFDA. After 8h treatment with Ad-mock, Ad-vp3, and Ad-vt, the cells were washed with PBS three times and stained with CM-H2DCFDA (10µM) for 30 min at 37 °C with shaking. The level of ROS was measured by a BECKMAN COULTER flow cytometer.

### LDH release assay

The signature indicator of pyrotosis, LDH, was detected by the CytoTox 96 Non-Radioactive Cytotoxicity Assay Kit (Promega) according to the manufacturer's instructions. The absorbance was measured at 450 nm with a universal microplate reader.

### SiRNA knockdown

To achieve the knockdown of the target protein, HCT116 cells were plated into sox-well plates (4×10^5^ per well). GSDME (RiboBio), GSDMD (RiboBio), caspase-3 (CST), caspase-9 (RiboBio), bax (RiboBio), Tom20 (RiboBio) siRNAs, and a siRNA negative control (RiboBio), were transfected with RNAiMAX reagent (Invitrogen) according to the manufacturer's instructions. After 48h, HCT116 cells were treated with the recombinant oncolytic adenoviruses for the follow-up experiment.

### JC-1 staining assay

To detect the trends of mitochondrial membrane potential, the JC-1 staining was performed. Cells were plated on a 6-well plate as described above and treated with Ad-mock, Ad-vp3 and Ad-vt for 36 h. The cells were then stained with the JC-1 staining solution (1×) for 15 min and observed under fluorescent microscope using 488 nm and 568 nm filters.

### Mouse models

Female nude mice (BALB/c, 4-5 weeks old) were fed with SPF-grade sterilized rat diet and water in a sterile environment. Animals were subjected to adaptive feeding for 7-10 days.

To conduct the xenograft tumor model, mice were subcutaneously injected with 100μl (5 × 10^6^ cells/mL) of colon cancer cells into the right side of the thigh. Four days after inoculation, the nude mice were randomly divided into four groups, namely PBS, Ad-mock, Ad-vp3 and Ad-vt. The tumors' size was measured every three days using an electronic digital caliper. The tumors volume was calculated as the following: Tumor volume = a × b^2^ × 0.5 (a = tumor length; b =short diameter of the tumor). The corresponding purified recombinant oncolytic adenovirus was injected into the tumor every three days (5×10^7^ PFU/100μl /intratumor injection). The relative inhibition rate of tumor growth was calculated, and an average tumor inhibition curve was plotted. Survival was recorded every day for six weeks. A graph indicating survival time (in days) vs. survival rate was further plotted.

For the luminescence imaging experiments, 5 × 10^6^ luciferase-expressing HTC116 cells were suspended in PBS and subsequently injected into the right side of the thigh with a volume of 100 μL. The described treatments above were performed by intratumoral injection every three days for six weeks. The results were detected using an IVIS@ Lumina S5 system 15 min after intraperitoneally injecting 3 mg D-luciferin (15 mg/mL in PBS).

### Statistical analysis

The statistical analysis was conducted using data from at least three independent experiments using SPSS 20.0 (SPSS Inc., Chicago, IL, USA). The results were statistically analyzed by Student's t-test or one-way analysis of variance (ANOVA; p < 0.05). Multiple comparisons were performed using the Student-Newman-Keuls test. P < 0.05 was considered as statistically significant.

## Results

### The effects of apoptin on cells growth

**Figure 1:**
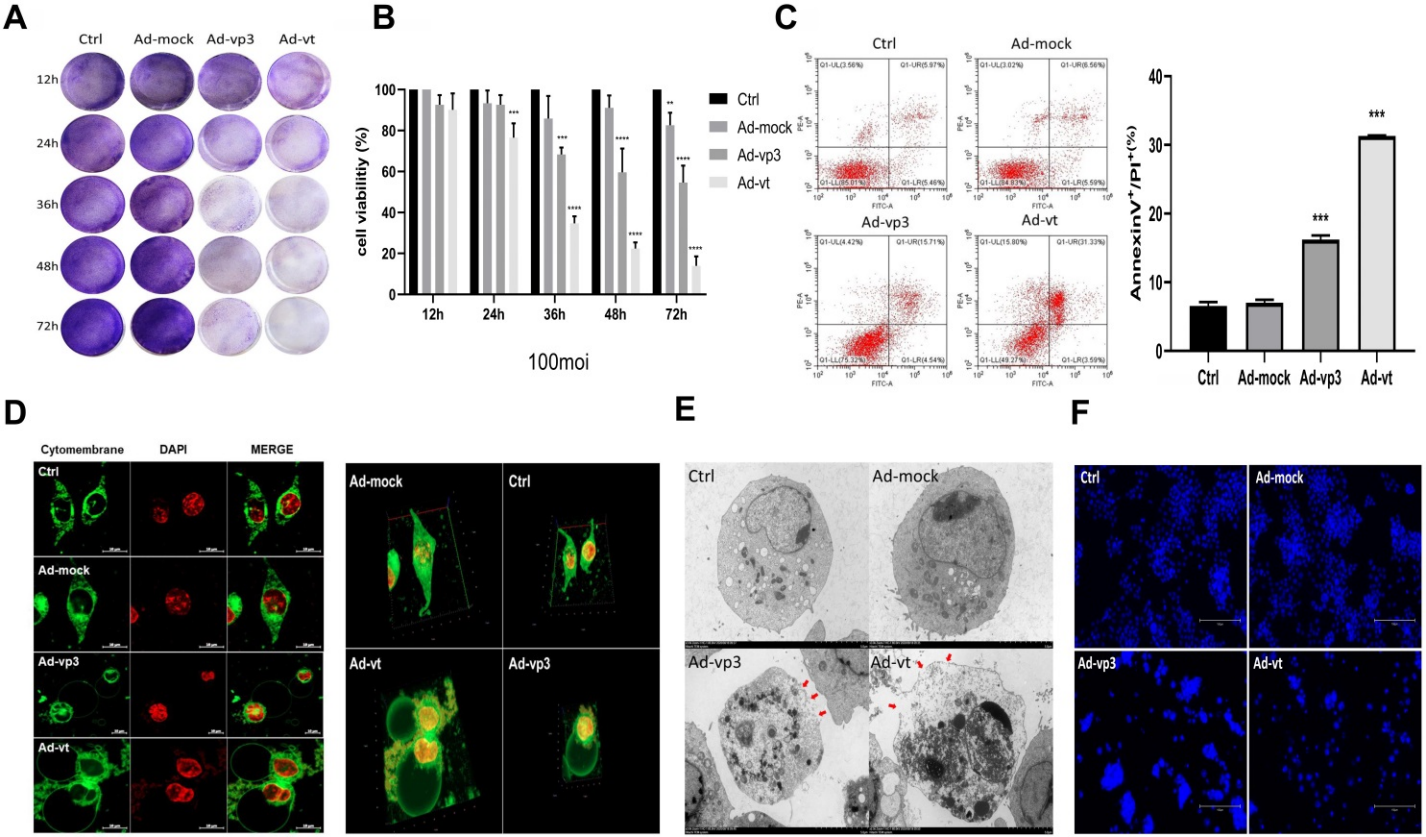
Effect of apoptin on cell membrane and inhibition of HCT116 cellviability. (A) HCT116 cells were treated with Ad-mock, Ad-vp3 and Ad-vt at 100 MOI, and with crystal violet staining at 12, 24, 36, 48, 72 hours. (B) HCT116 cells were treated with Ad-mock, Ad-vp3 and Ad-vt at 100 MOI, the viability of cells was determined by the CCK8 assay. (C) The percentage of apoptosis (stained with Annexin V^+^/PI^-^) or pyrotosis (stained with Annexin V^+^/PI^+^) was indicated at 36h. (D) A laser confocal microscope image (E) transmission electron microscopy. The red arrowheads indicate the emerging pore from the plasma membrane. (F) Hoechst staining of cells treated with Ad-vp3 and Ad-mock respectively showed the proportion of nuclear thickening and nuclear fragmentation.

More than 70 types of tumor cells have been reported to be sensitive to apoptin cell death-mediated effect, including melanoma, lymphoma, colon, breast, and lung cancers. However, the mechanism of its effect on tumor cells is not well understood 4. To test the cell viability, we treated HCT116 cells with recombinant adenovirus expressing apoptin (Ad-vp3) and adenovirus expressing apoptin-hTERTp (Ad-vt) at a dose of 10 MOI and 100 MOI for 12h, 24h, 36h, 48h, and 72h, and performed crystal violet staining and CCK8 analysis (Fig. 1A and B, Supplementary Fig. 1A). The crystal violet experiment showed that Ad-vp3 and Ad-vt had obvious killing effects on cells, which was also confirmed by CCK8 experiment, and showed that the killing effect of Ad-vt was stronger than that of Ad-vp3. These results indicate that Ad-vp3, and Ad-vt have significant time-dependent effects, while Ad-mock had almost no effect on cells.

### Effects of apoptin on cell morphology and plasma membrane

**Figure 2:**
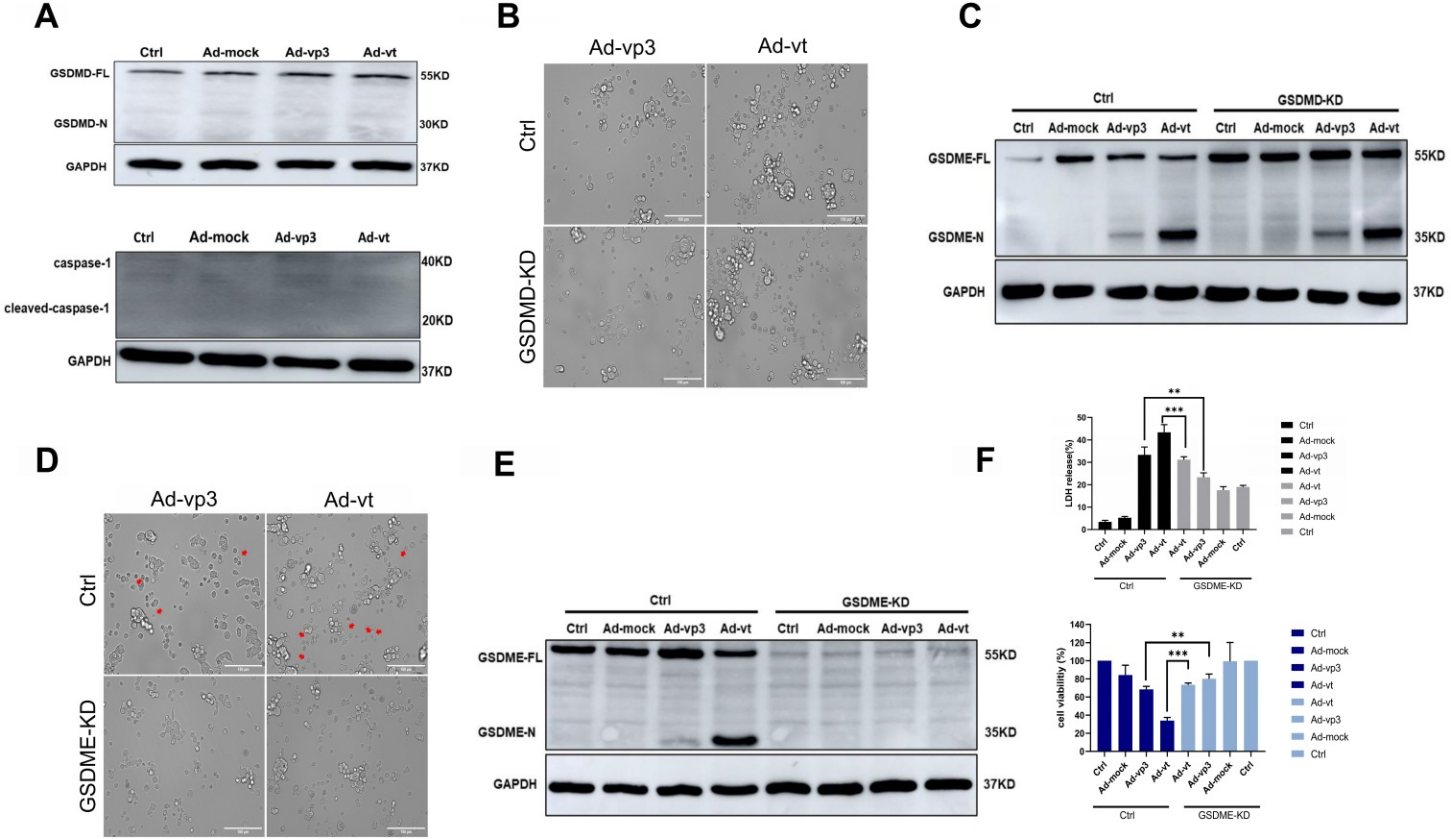
Apoptin-induced pyroptosis by GSDME rather than GSDMD. (A) Expression of GSDMD and capase-1. HCT116 cells were treated with Ad-mock, Ad-vp3 and Ad-vt at 100 MOI, (B) microscopic imaging and (C) cleavage of GSDME were performed in the presence or absence of GSDMD knockdown. Microscopic imaging (D) and (E)cleavage of GSDME were performed in the presence or absence of GSDME knockdown. (F) Knockdown of GSDME diminished LDH release and increased viability of HCT116 cells following treatments with Ad-vp3 and Ad-vt. Data are shown as mean ± SD (*p < 0.05, **p < 0.01, ***p < 0.001)

The annexin V-FITC/PI assays showed that Ad-vp3 and Ad-vt can increase the percentage of double-positive cells (Fig. 1C). However, Annexin V-FITC/PI assays are a semi-quantitative experiment, and the increase in the ratio of double positive cells did not confirm the occurrence of pyroptosis. As shown in Fig. 1F, the Hoechst assay shows that there is a small amount of nuclear fragmentation when the HCT116 cells were treated with Ad-vp3 and Ad-vt. However, nuclear fragmentation cannot be used as a marker to distinguish between the pyroptosis and apoptosis. Therefore, we stained the cell membranes for 36h and observed them under a laser scanning confocal microscope. As shown in 2D or 3D images (Fig. 1D), HCT116 cells treated with Ad-vp3 and Ad-vt showed swelling large bubbles appeared in the plasma membrane of swollen cells. This is obviously different from the classical morphology of apoptotic cells. Moreover, using transmission electron microscopy (TME), it was found that several pores were formed on the cell membrane (Fig. 1E). In addition, the experimental results of LDH release showed that the LDH release of HCT116 cells treated with Ad- vp3 and Ad-vt increased significantly (Fig. 2F), further indicating the perforation and rupture of plasma membrane. Taken together, these results suggest that Ad-vp3 and Ad-vt may induce HCT116 cell death through pyroptosis.

### Apoptotin induces pyroptosis via GSDME

Gasdermins are a family of proteins that are involved in the execution of the necrotic cell death program. Among these, GSDMD and GSDME have been reported to trigger the execution of pyropotis 15, 16. Viruses are membrane-damaging agents that can activate NLRP3 inflammasomes, which binding to the apoptosis-associated speck like protein for caspase-1, lead to GSDMD cleavage 27. To confirm whether GSDMD is involved in apoptin-induced pyroptosis, we investigated its expression in HCT116 cells treated with Ad-vp3 and Ad-vt and found that GSDMD was not cleaved. We also observed that Caspase-1, an upstream protein of GSDMD was not expressed (Fig. 2A). Moreover, GSDMD knockdown did not significantly reduce the number of cells with pyroptosis morphological characteristics (Fig. 2B) and had no effect on the cleavage of GSDME (Fig. 2C). Therefore, these experiments confirmed that GSDMD neither plays an inhibitory effect nor participates in apoptin-induced pyroptosis.

The N-terminal (N-GSDM) domain of GSDME is a marker of proptosis and that has a perforating activity which results in cell death via pyroptosis 28. GSDME knockdown decreased the number of cells with pyroptosis morphological characteristics (Fig. 2D). Furthermore, release of LDH was remarkably attenuated (Fig. 2F), and cleavage of GSDME was completely reversed (Fig. 2E). To determine whether adenoviral replication is involved in apoptin-induced pyroptosis, the possibility that Ad-T (Ad5-hTERT-E1a) may cause pyroptosis was tested. Western blot results showed that Ad-T cannot induce the expression of GSDME N-terminus (Supplementary Fig. 1D). Western blot analysis was performed when the nine different types of cancer cells were treated with Ad-vp3. Only HepG2 and 446 cancer cells had very few GSDME cleavage protein bands (Supplementary Fig. 1E), while the other seven cells did not undergo pyroptosis. There may also be necroptosis with the characteristic morphological features of pyroptosis under the microscope, so we use the necroptosis inhibitor GSK^'^872 to distinguish pyroptosis and necroptosis. As shown in Supplementary Fig. 1B, GSK'872 did not significantly reduce the number of cells with pyroptosis morphological characteristics and did not affect expression status of GSDME N-terminus (Supplementary Fig. 1C). In summary, our results show that apoptin induces pyroptosis in HCT116 cells by cleaving GSDME.

### Apoptin-induced pyroptosis is dependent on the caspase family of proteases

**Figure 3:**
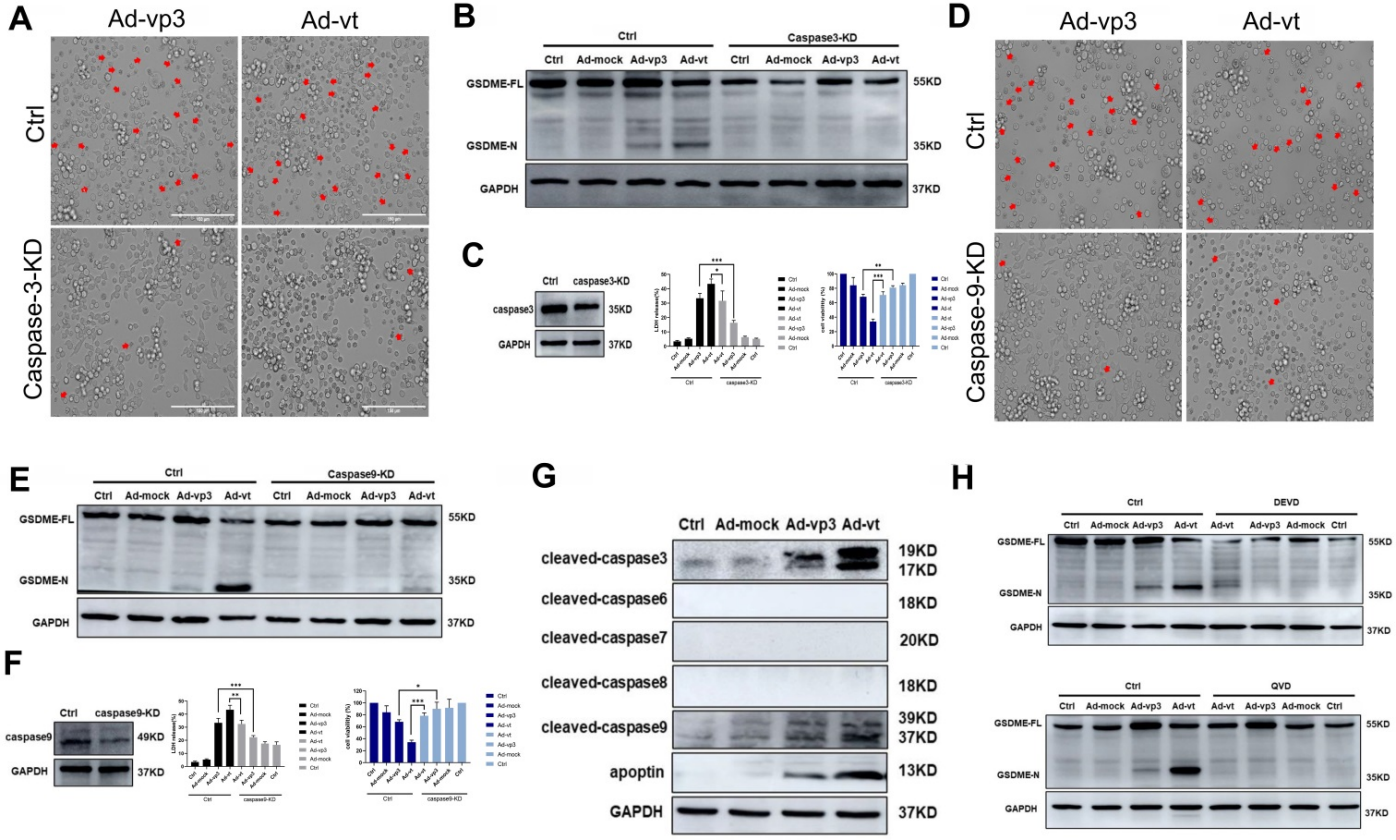
Apoptin-induced pyroptosis depends on the caspase family of proteases. (A) HCT116 cells were treated with siRNA targeting caspase-3. (B) Cleavage of GSDME was detected by western blot. (C) GSDME knockdown diminished LDH release and increased HCT116 cellsviability. (D) HCT116 cells were treated with siRNA targeting caspase-9. (E) GSDME cleavage was detected by western blotting. (F) GSDME knockdown diminished LDH release and increased HCT116 cells viability. The red arrowheads indicate characteristics of pyroptotic cells. (G) Expression of cleaved-caspase-3, cleaved-caspase-6, cleaved-caspase-7, cleaved-caspase-8, cleaved-caspase-9, and apoptin. (H) Pretreatment with caspase-3 specific inhibitor DEVD and caspase broad-spectrum inhibitor QVD. GSDME was detected by western blotting. Data are shown as mean ± SD (*p < 0.05, **p < 0.01, ***p < 0.001)

Caspases are cysteine proteases that cleave their target proteins at the site of aspartic acid residues. Programmed cell death, such as apoptosis, necrosis and pyroptosis, require the involvement of caspases 27. To identify which caspases may be involved in apoptin-induced pyroptosis, the levels of active caspase-3/-6/-7/-8/-9 were investigated in response to Ad-vp3 and Ad-vt treatment (Fig. 3G). The results showed that vp3 and Ad-vt enhanced the expression of cleaved-caspase-3 and cleaved-caspase-9, while caspase-6/-7/-8 was not activated. Therefore apoptin-induced pyroptosis may depend on the caspase pathway.

To further investigate this, we first verified whether active caspase-3 was necessary for apoptin-induced pyroptosis. We used siRNA technology to knockdown the expression of caspase-3. The number of pyroptosis cells was significantly reduced in caspase-3 knockdown cells when observed under the microscope (Fig. 3A). LDH release was also considerably decreased (Fig. 3B). Additionally, caspase-3 knockdown completely blocked the cleavage of GSDME (Fig. 3C). The results show that activated caspase-3 cleaves GSDME.

Second, we explored the involvement of active caspase-9 in apoptin-triggered pyroptosis. Knockdown of caspase-9 resulted in the blockage of most GSDME cleavage, LDH release, and pyroptotic cell when examined under the microscope (Fig. 3D, 3E and 3F). The results suggested that the cleavage of GSDME depended on the activated caspase-9 and caspase-3. Furthermore, HCT116 cells pre-treatment with the caspase-3-specific inhibitor zDEVD-FMK and the non-specific caspase inhibitor QVD-FMK (Fig. 3H), resulted in zDEVD-FMK inhibition of GSDME cleavage, while QVD-FMK reversed its cleavage. Taken together, these results confirm that apoptin-induced cell pyroptosis depends on activated caspase-3 and caspase-9.

### Apoptin-activated GSDME-dependent pyroptosis through the mitochondrial intrinsic apoptotic pathway

**Figure 4:**
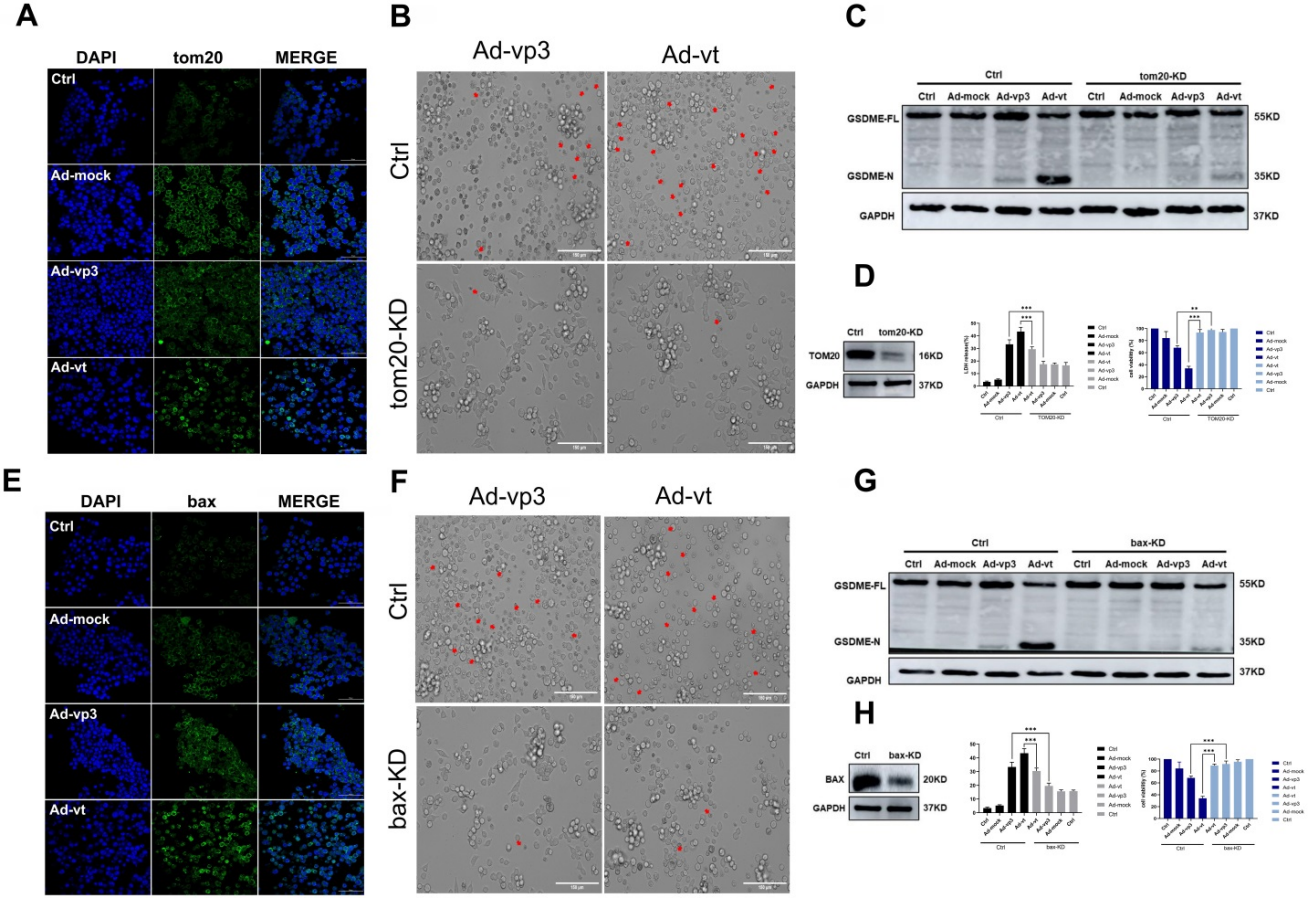
Apoptin-activated GSDME-dependent pyroptosis through the mitochondrial intrinsic apoptotic pathway. (A) Ad-vp3 and Ad-vt induced Tom20 accumulation and aggregation towards the nucleus. (B) HCT116 cells were treated with siRNA targeting Tom20. (C) Cleavage of GSDME was detected by western blot, (D) Knockdown of Tom20 diminished LDH release and increased viability of HCT116 cells. (E) Ad-vp3 and Ad-vt induced bax accumulation and aggregation in the nucleus. (F) HCT116 cells treated with bax siRNA targeting. (G) GSDME cleavage was detected by western blot. (H) Bax knockdown diminished LDH release and increased HCT116 cells' viability. Data are shown as mean ± SD (*p <0.05, **p <0.01, ***p <0.001)

**Figure 5:**
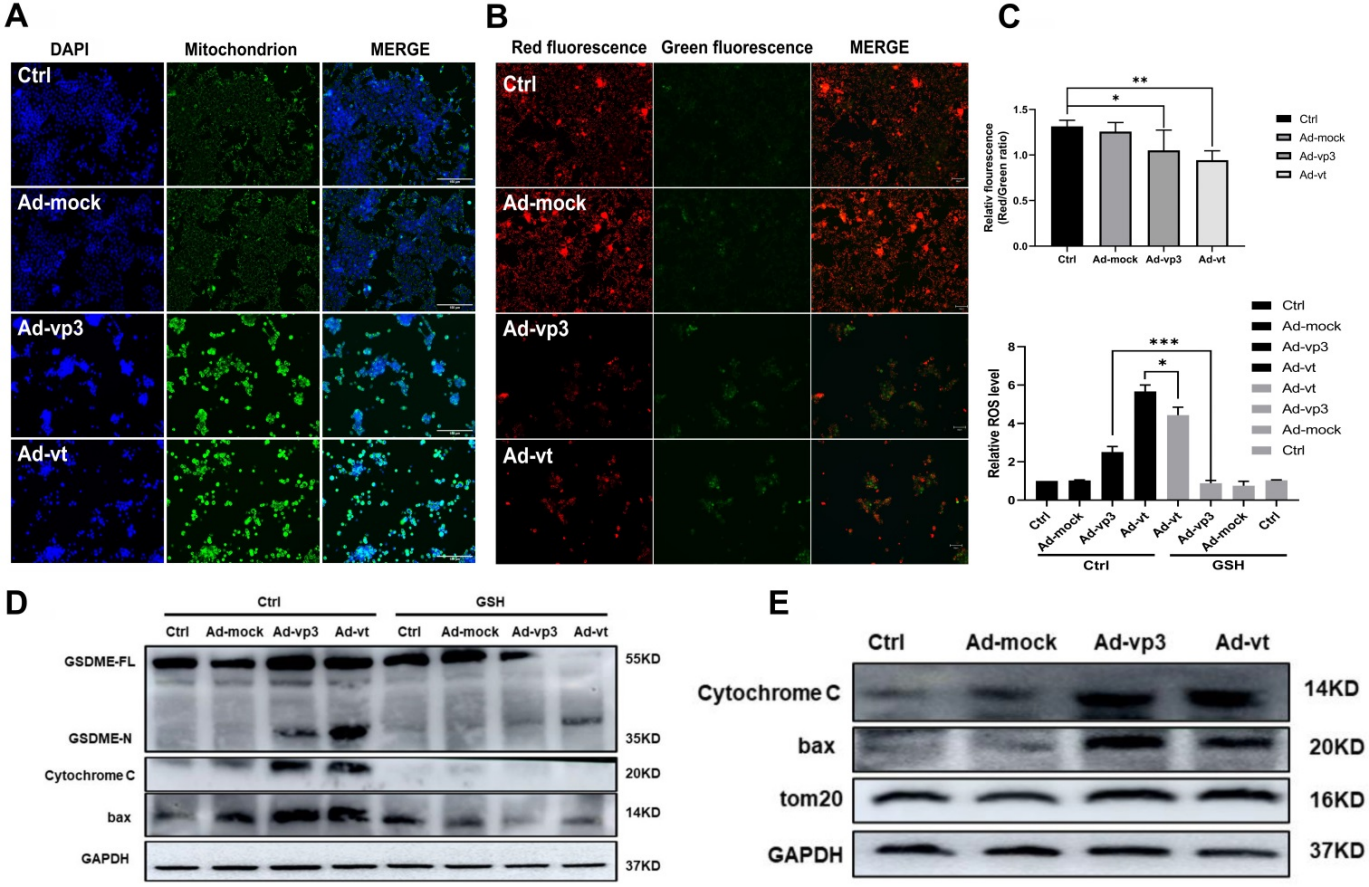
Apoptin induced pyroptosis through the ROS pathway. (A) Ad-vp3 and Ad-vt caused mitochondria aggregation towards the nucleus. (B) Red fluorescence changes to green fluorescence, measured by fluorescence microscopy following JC-1 staining. The ratio of red fluorescence to green fluorescence decreased. (C) Measurement of ROS levels. (D) The expression of cleaved-GSDME, BAX, and cytochrome c in HCT116 cells was analyzed by flow cytometry in the presence or absence of GSH. (E) Detection of the expression of bax, cytochrome c, Tom20 by western blotting. Data are shown as mean ± SD (*p < 0.05, **p < 0.01, ***p < 0.001)

It has been reported that apoptin triggers apoptosis via the classical mitochondrial intrinsic pathway 29, 30, and independently of the receptor extrinsic pathway. To determine whether mitochondria are involved in apoptin-induced pyroptosis, we first performed a mitochdrial staining on cells that were treated with Ad-vp3 and Ad-vt and observed them under the microscope (Fig. 5A). We found that in the treated cells, the mitochondria significantly agglomerate towards the nucleus. Next, the JC-1 staining was performed to detect the change of mitochondrial membrane potential (MMP) (Fig. 5B). Cells treated with Ad-vp3 and Ad-vt caused an imbalance and disequilibrium of MMP as shown by the red aggregates to green monomers and the reduction of the ratio red fluorescence/green fluorescence. According to reports, Tom20 is very important for bax mitochondrial localization and promotion of cytochrome C release 31. Western blot (Fig. 5E) and laser scanning confocal microscopy (Fig. 4A) showed a significant increase in Tom20 expression. Therefore, we investigated whether bax is involved in Ad-vp3- and Ad-vt-induced pyroptosis. Ad-vp3 and Ad-vt treatments caused bax to move towards the nucleus where it accumulated and multiplied (Fig. 4E). This is consistent with the observed accumulation of mitochondria into the nucleus. Bax knockdown not only led to a significant reduction in the number of pyroptotic cells (Fig. 4F) and an effective inhibition of cleavage of GSDME (Fig. 4G), but also suppressed LDH release and cell death (Fig. 4H). Therefore, bax is required for apoptin-induced pyroptosis. In addition, bax also acts as an upstream factor of activated caspase-9 that was increased at different degrees (Fig. 5E). Thus, these and previous results confirm that apoptin induces GSDME-dependent pyroptosis via the mitochondrial intrinsic apoptotic pathway.

### Apoptin induced pyroptosis through the ROS pathway

It has been reported that apoptin can significantly inhibit the growth of liver cancer cells by causing cell apoptosis 11. We also want to explore whether ROS are involved in apoptin induced pyroptosis. The endogenous ROS levels of cells treated with Ad-vp3 and Ad-vt were detected by flow cytometry (Fig. 5C). The results showed that Ad-mock group had no effect on the level of ROS, while the level of intracellular ROS increased in Ad-vp3 and Ad-vt groups. GSH (glutathione) as a ROS scavenger can attenuate the level of ROS induced by Apoptin. Correspondingly, GSH blocks mostly bax, cytochrome c and cleavage of GSDME (Fig. 5D).

It has been reported that Tom20 can sense and transmit ROS signal to mitochondria 31. Therefore, the localization of Tom20 on the surface of mitochondrial membrane may be involved in apoptin-induced pyroptosis that is associated with cytochrome C and bax driven release from mitochondria to cytosol by Ad-vp3 and Ad-vt. The results of laser confocal microscopy showed that Tom20 enhances and gathers toward the nucleus, which is consistent with the results of mitochondrial staining. Ad-vp3 and Ad-vt treatment induced GSDME cleavage (Fig. 4C), LDH release (Fig. 4D), pyroptotic cells (Fig. 4B). Knockdown of Tom20 greatly weakened these effects and cell death was almost completely reversed. These results demonstrate that apoptin induces pyroptosis through the ROS pathway.

### The interaction between apoptosis and pyroptosis is induced by apoptin

**Figure 6:**
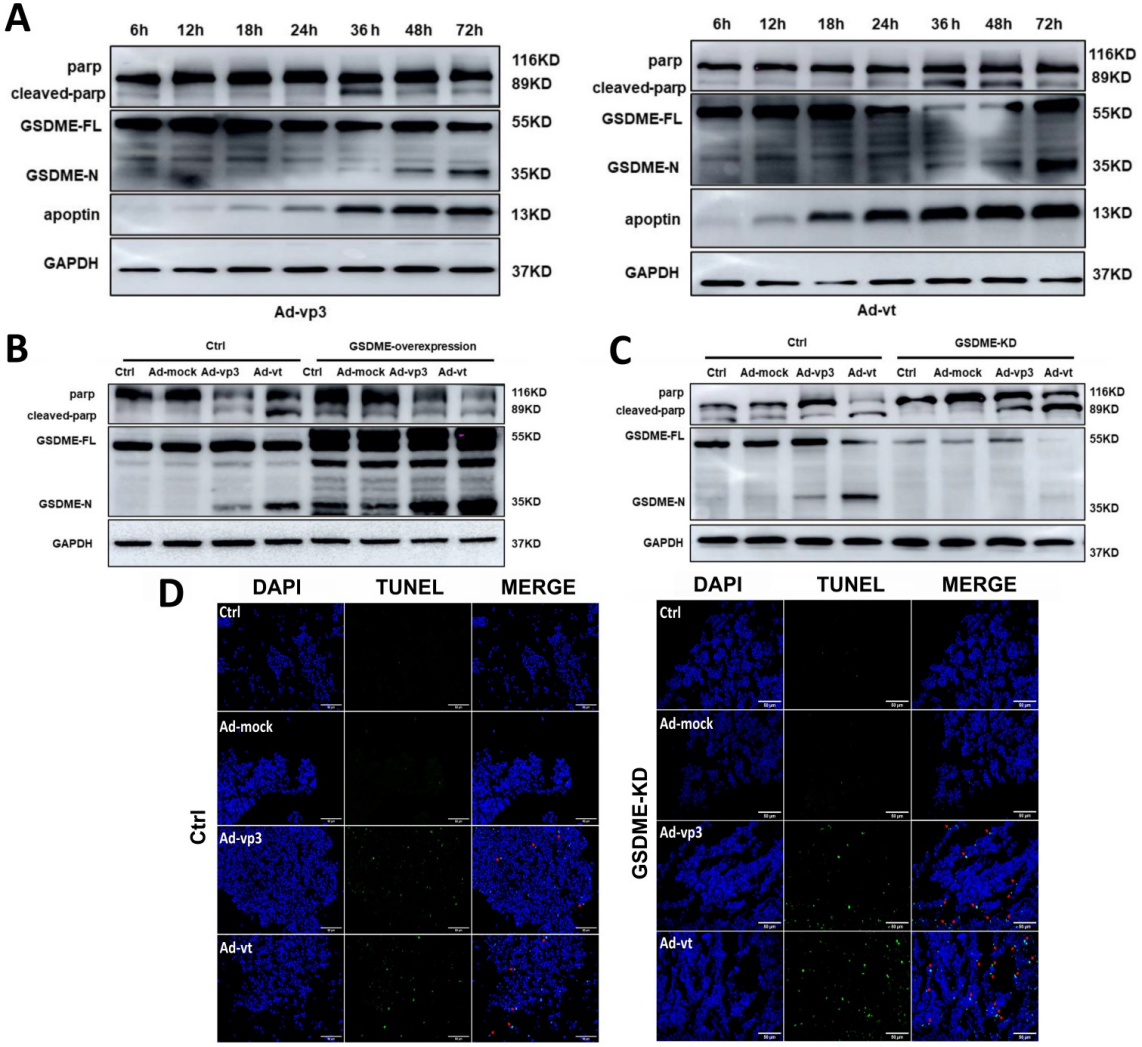
The interaction between apoptosis and pyroptosis induced by apoptin. (A) HCT116 cells were treated with Ad-vp3 and Ad-vt for 6h to 72h. The markers of pyroptosis and apoptosis were analyzed by western blot. (B) Western blot analysis of markers of pyroptosis and apoptosis with or without GSDME overexpression. (C) In the presence or absence of GSDME knockdown. (D) The quantification of positive apoptotic cells was assessed by the TUNEL assay, with or without GSDME knockdown.

According to the abovementioned experimental results, apoptin-induced pyroptosis and apoptosis share the same regulatory mechanisms. Thus, further investigations are needed to explore the mechanism by which pyroptosis and apoptosis cooperate and interact with each other in cancer cells. Due to the formation of pores in the cell membrane, the integrity of the plasma membrane is lost leading to a typical pyroptotic death. GSDME-mediated pyroptosis was considered to be a secondary necrosis 17. Other studies have suggested that chemotherapy-mediated pyroptosis occurs earlier than apoptosis. Treatment with the apoptotic agonist staurosporine (STS) can distinguish apoptosis from pyroptosis (Supplementary Fig. 1F). However, STS can only and specifically cause PARP breakage rather than GSDME cleavage. In the next experiment, we probed the senescence marker protein of apoptosis and pyroptosis from 12h to 72h (Fig. 6A). The experiment results showed that cleavage of GSDME and cleavage of PARP first appears 36 hours after treatments and lasts until 72 hours. It is suggested that apoptin induced pyroptosis occurs simultaneously with apoptosis.

Therefore, it is reasonable to believe that there may be some specific regulatory mechanism between apoptin-induced pyroptosis and apoptosis. We found that GSDME knockdown leads to more cleavage of PARP (Fig. 6C). To further explore the effect of GSDME expression on apoptosis, TUNEL assay was performed, and it was observed that apoptosis increased in GSDME knockdown cells, which was consistent with the western blot results (Fig. 6D). The results indicated that GSDME knockdown switches apoptin-induced cell death from pyroptosis to apoptosis. By contrast, GSDME overexpression relatively attenuated the cleavage of PARP (Fig. 6B). Although there are complex regulatory mechanisms, our results suggest that pyroptosis and apoptosis co-occur and interact with each other. It is considered that apoptin caused apoptosis and pyroptosis through the same upstream pathway; however, the crosstalk between apoptosis and pyroptosis is still complex.

### Inhibitory effect of apoptin and induction of pyroptosis *in vivo*

**Figure 7:**
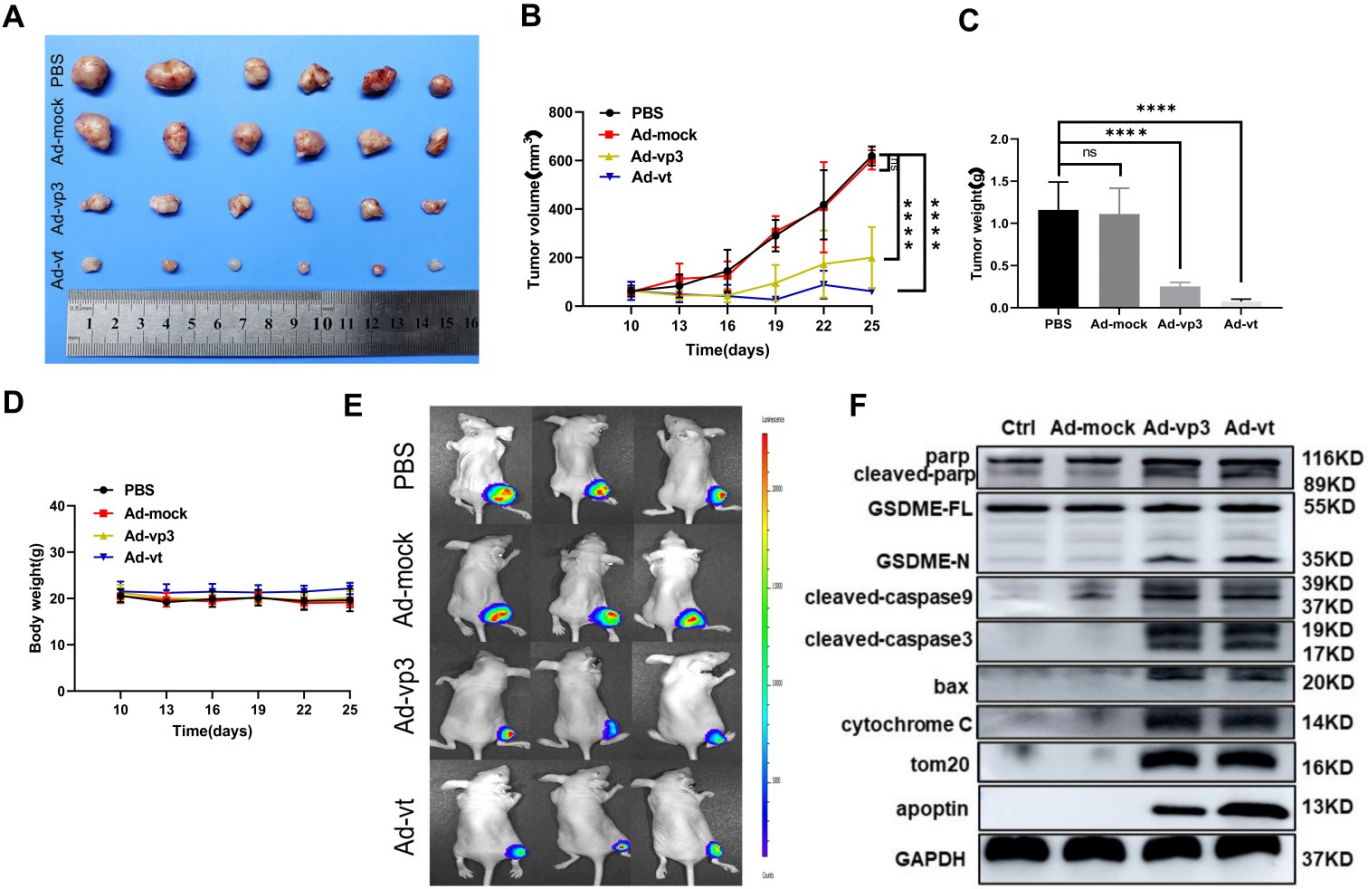
*In vivo* effect of apoptin in suppressing colon cancer tumor growth. HCT116 cells were subcutaneously injected into the right hind limb of nude mice (n=6). After seven days, Ad-mock, Ad-vp3 and Ad-vt were injected into the tumors every three day for 3 weeks. (A) Image of xenograft tumors, (B) tumor volume. (C) tumor weight and (D) body weight, were recorded at the indicated times. (E) Tumor metastasis was quantified using bioluminescence imaging. (F) The cleavages of PARP, GSDME, caspase-3, caspase-9, Bax release, cytochrome c, and Tom20 were detected from the tumor samples.

To explore the apoptin-induced cell death *in vivo*, HCT116 and luciferase-expressing HTC116 cells were subcutaneously injected into nude mice that were also administered with Ad-mock, Ad-vp3, and Ad-vt. Ad-mock treatment showed no effect on tumor growth, while the treatment of Ad-vp3 and Ad-vt significantly reduced the volume and weight of tumors respectively (Fig. 7A, B and C). The average body weight of the experimental group did not significantly decrease compared with the control group (Fig. 7D). In addition, we also performed *in vivo* imaging to detect the fluorescence of HCT116 cells. Consistent with the previous experimental results, the fluorescence of the experimental group was significantly attenuated compared to the control group (Fig. 7E). Furthermore, we tested whether apoptin-induced cell death *in vivo* was associated with pyroptosis. Therefore, we detected several protein levels of the mitochondrial pathways in the above experiments. Consistent with previous *in vitro* experiments, Ad-vp3 and Ad-vt treatment increased the levels of cleaved-PARP, cleaved-GSDME, cleaved-caspase-3, cleaved-caspase-9, and up-regulated the expression of bax and cytochrome C. In summary, apoptin can inhibit tumor growth and induce pyroptosis *in vivo*.

## Discussion

Colorectal cancer (CRC) is the fourth most common cancer worldwide, accounting for eleven percent of all diagnosed cases and the second most leading cause of death 32, 33. At present, surgical treatment and chemotherapy are the main methods to treat colon cancer. Nevertheless, more than 50% of patients with colorectal cancer often miss the best time for diagnosis and treatment, and the 5-year survival rate is less than 33%. Therefore, there is an urgent need to develop a promising treatment for CRC.

Adenoviruses are popular gene delivery vectors that can effectively express the inserted exogenous genes to infect both dividing and non-dividing cells 34. Currently, oncolytic adenoviruses have been reported to be a promising approach in treating diverse tumor types 35. In our study, we used human adenovirus type 5 (Ad5) as a carrier to construct a recombinant adenovirus Ad-vp3 expressing apoptin. Another recombinant adenovirus Ad-vt was constructed to specifically replicate to express more apoptin. These two recombinant oncolytic adenoviruses were demonstrated to efficiently yield apoptin causing cell death *in vitro* 36. The Crystal Violet and cell viability assays demonstrated that apoptin significantly kills HCT116 cancer cells. Additionally, the results of *in vitro* tumor inhibition showed that treatments with Ad-vp3 and Ad-vt could also inhibit HCT116 tumor growth. Therefore, apoptin could be used as a potential therapeutic bullet in antitumor therapies in the future.

We observed typical characteristics of pyrotosic cells under the microscope, such as cell swelling, and large bubbles appearing on the HCT116 cells, indicating that the integrity of cell membrane is damaged due to pore formation. In addition, our results showed some characteristic of pyroptosis, such as increase of LDH, the ratio of FITC^+^/PI^+^ positive cell, and GSDME cleavage. At the same time, we also observed cell necroptosis, a form of PCD, which showed similar characteristics to pyroptosis, such as membrane disruption and cell swelling. We used the necroptosis inhibitors GSK'872 to distinguish between the two processes and showed that GSK'872 neither reduced the number of pyroptosic cells nor affected cleavage of GSDME. Thus, this result demonstrated that apoptin-induced cell death was not associated with necroptosis.

It has been reported that apoptin induces cell death in diverse human cancer cell lines 37. Apoptin can cause cancer cell apoptosis independently of p53 38. Although there is some evidence showing that apoptin triggers cell death in association with caspases, the mechanisms of apoptin-induced cell death are still unclear. In this study, we extend the conventional concept of apoptin and demonstrate that apoptin can induce GSDME-mediated pyrotosis via the mitochondrial intrinsic apoptotic pathway.

We detected GSDME protein expression in three other CRC cell lines and showed that its protein expression greatly varies in these three types of cells. The sw-620 and sw-460 cells hardly expressed GSDME, therefore, they do not have the ability to produce enough GSDME-NL to perform pyroptosis. The caco2 cells expressed a certain amount of protein, but did not show characteristics of pyroptosis. On one hand, the cells are probably not sensitive to apoptin, and on the other hand, they are not sensitive to adenovirus, so they can not translate enough apoptin to effectively activate caspase-3. Finally, GSDME cannot be cut to induce pyroptosis. Therefore, the induction of pyroptosis by apoptin in colon cancer cells is not a universal mechanism.

For a long time, pyroptosis has been considered to be associated with the caspase-1-dependent classical pathway and caspase-4/-5/-11 dependent non-classical pathway. GSDMD is cleaved by activated caspase-1/4/5/11 to form pores in the plasma membrane leading to pyroptosis. It has been reported that there are various membrane-damaging agents that can trigger pyroptosis via the canonical inflammasome-pathway, such as viruses, bacteria, and danger associated molecular patterns (DAMPs) 39. Therefore, we speculated that recombinant oncolytic adenoviruses also induced pyroptosis via GSDMD. However, GSDMD was expressed at low level in HCT116 cells, and we did not detect its cleavage. Knockdown of GSDMD did not significantly reduce the number of pyroptosic cells either. As a result, GSDMD does not participate in apoptin-induced pyroptosis.

So far, the Gasdermin family of proteins mainly consists of six members: GSDMA, GSDMB, GSDMC, GSDMD, GSDME, and DFNB58. Among them, GSDME has been widely investigated for its involvement in pyroptosis of cancer cells. Normally, GSDME is highly expressed in normal tissues, and in certain cancer lines, such as melanoma, lung breast, colon, and esophageal cancers 40. Researchers found that GSDME possess caspase-3-cleavable site267DMPD270 16, and that GSDME-dependent pyroptosis can be induced by the mitochondrial apoptotic pathway 41, 42. Our results were consistent with previous studies, in which we showed that treatments with Ad-vp3 and Ad-vt lead to mitochondria aggregation towards the nucleus and a decrease in the activation of MMP; bax; cytochrome C; caspase-9; caspase-3 and cleavage of GSDME. Apoptin-induced pyroptosis is also significantly attenuated by siRNA knockdowns of caspase-3 and caspase-9. Moreover, QVD, a pan caspase inhibitor, could reverse the pyroptosic phenomenon, which confirmed that apoptin-induced pyroptosis via the mitochondrial intrinsic apoptotic pathway is highly dependent on caspases.

It has been reported that ROS in cancer cells play a critical role in mediating and regulating apoptosis, which modulates tumor cells' proliferation 43. ROS can trigger the oxidation and oligomerization of the mitochondrial membrane protein Tom20, leading to pyroptosis through the mitochondrial pathway. These observations are consistent with previous results. Our data show that treatments with Ad-vp3 and Ad-vt can boost the level of ROS and activate tom20-bax-caspase-GSDME mediated pyroptotic cell death. However, GSH can attenuate the release of bax and cytochrome C. Finally, cleavage of GSDME is also blocked by GSH, demonstrating that ROS is an upstream signal that regulates pyrotosis via the mitochondrial pathway. Our data suggest that the mitochondrial pathway mediated apoptosis and pyrotosis are simultaneously involved in the context of apoptin treatment. As a result of cell death, the differential expression of GSDME and caspase-3-cleavable sites on downstream effectors may determine the order of these two processes and the terminal form of cell death related to caspase-3 activity. We have some reasons to speculate that apoptin induced pyrotosis is a major cause of cell death in HCT116 cells. Pyrotosis alone is sufficient to cause cell death, while GSDME knockdown switches cell death from pyroptosis to apoptosis. Moreover, GSDME overexpression promotes GSDME cleavage concomitantly with the increase of cleaved-PARP expression. The correlation and internal transformation between apoptosis and pyrotosis have also been confirmed. We propose that apoptin triggers two types of PCDs to collaborate and transform each other in suppressing tumor growth.

Our study not only found a new mechanism of apoptin-induced cell death, but also opened a new way for future application of recombinant oncolytic adenoviruses. The signal transduction relationship between pyrotosis and apoptosis preliminarily demonstrated that these two processes closely work together to regulate cytotoxicity and inhibit tumor cells. Therefore, further study on the molecular mechanism of this interaction is of a great significance in the rational utilization of apoptin. In addition, it is still not clear how apoptin expression is precisely regulated. While it is increasingly clear that apoptin can sense the survival signal in tumor cells and transmit it to stimulate apoptosis and pyrotosis. At last, apoptin modulates the pattern of cell death and may contribute to the immunogenicity of tumor cells, which could serve as a theoretical basis for combining other treatment regimens. Given the above, apoptin is promising as a tumor-specific sensor and therapeutic agent in cancer therapy.

## Supplementary Material

Supplementary figure.

## Figures and Tables

**Figure 8:**
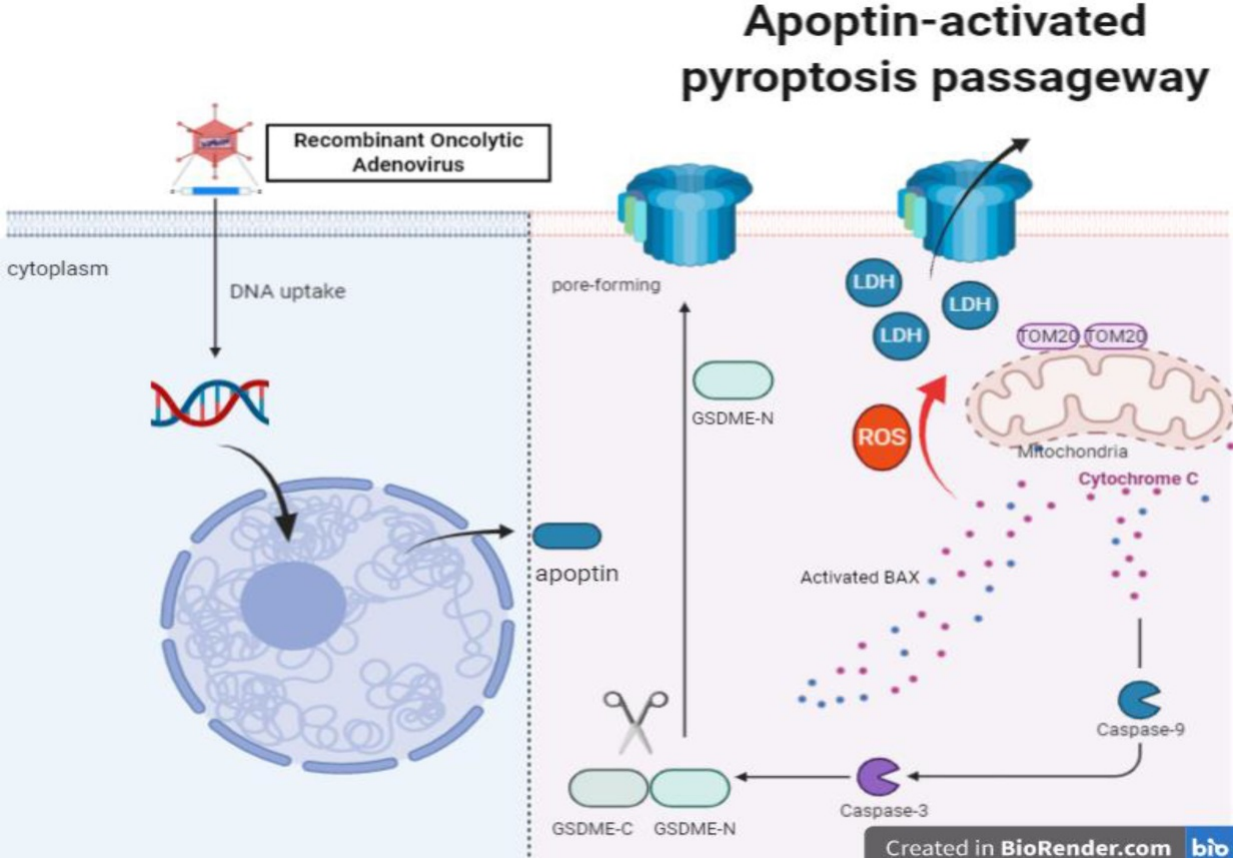
Apoptin enhanced ROS induced Tom20 increase in HCT116. Activation of bax and cytochrome c release activates caspase-9, which activates caspase-3. Ultimately, activated caspase-3 led to GSDME cleavage, then GSDME-N triggered pore-forming in cytomembranes and LDH release.
