# Supplementary material for: Apoptin induces pyroptosis of colorectal cancer cells via the GSDME-dependent pathway
Source: Int J Biol Sci. 2022 Jan 1;18(2):717–30. doi: 10.7150/ijbs.64350 (PMC8741846; doi:10.7150/ijbs.64350)
Supplement: Supplementary file 1 — Supplementary figure. [file ijbsv18p0717s1.pdf]

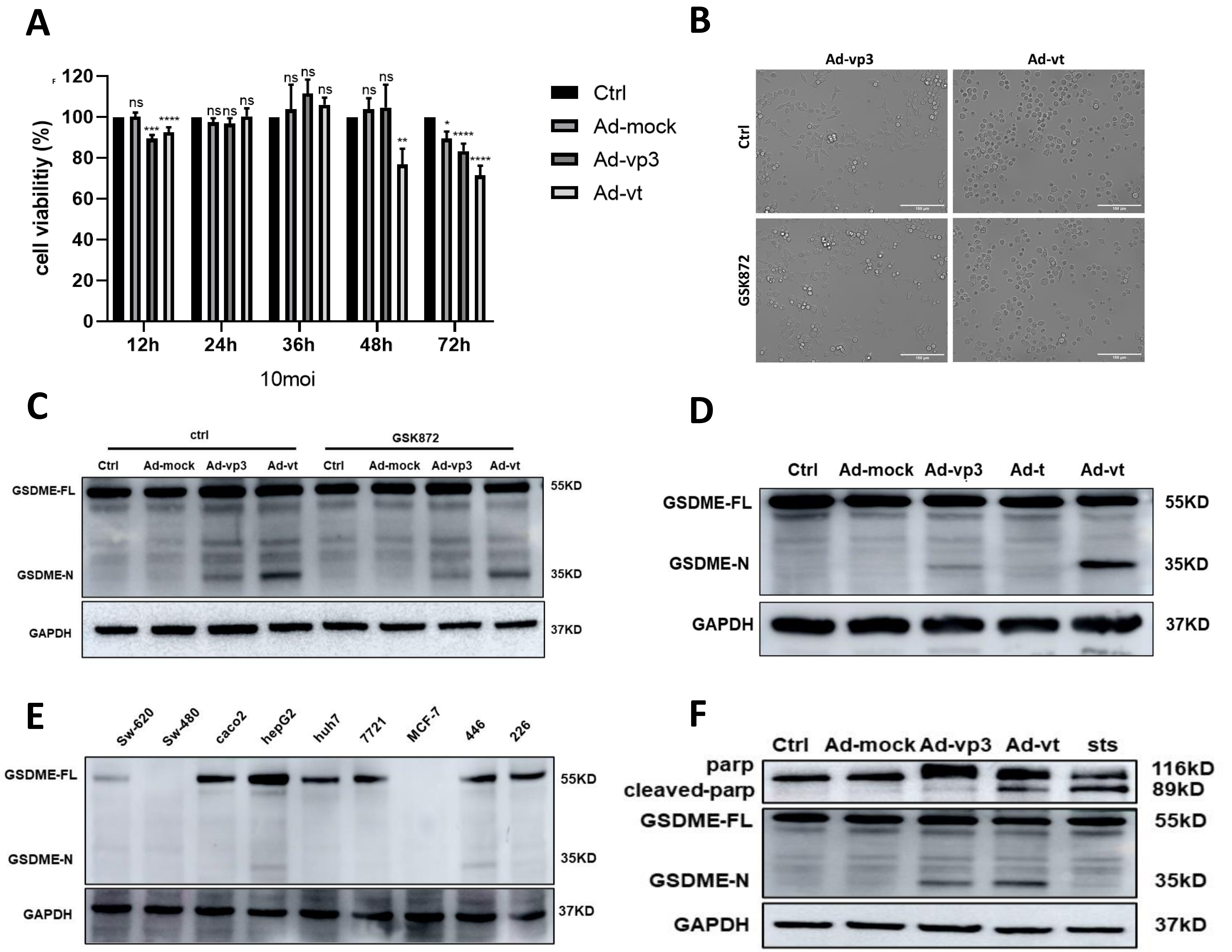

A
120
100
80
60
40
20
0
cell viabilitiy (%)
12h
24h
36h
48h
72h
10moi
ns
***
****
*
**
Ctrl
Ad-mock
Ad-vp3
Ad-vt
B
Ad-vp3
Ad-vt
Ctrl
GSK872
C
ctrl
GSK872
Ctrl
Ad-mock
Ad-vp3
Ad-vt
GSDME-FL
GSDME-N
GAPDH
55KD
35KD
37KD
D
Ctrl
Ad-mock
Ad-vp3
Ad-t
Ad-vt
GSDME-FL
GSDME-N
GAPDH
55KD
35KD
37KD
E
Sw-620
Sw-480
caco2
hepG2
huh7
7721
MCF-7
446
226
GSDME-FL
GSDME-N
GAPDH
55KD
35KD
37KD
F
Ctrl
Ad-mock
Ad-vp3
Ad-vt
sts
parp
cleaved-parp
GSDME-FL
GSDME-N
GAPDH
116kD
89kD
55kD
35kD
37kD

Supplementary Fig.1 (A) HCT116 cells were treated with 10 MOI of Ad-mock, Ad-vp3 and Ad-vt for 12, 24, 36, 48, 72 hours, and cell viability was determined by the CCK8 assay. (B) Image of pyroptotic cells with or without the necroptosis inhibitor GSK'872. (C) Cleavage of GSDME was detected by western blotting. (D) HCT116 cells were treated with Ad-mock, Ad-vp3, Ad-T and Ad-vt, followed by a detection GSDME cleavage. (E) Nine different types of cancer cells were treated with Ad-vp3, and GSDME cleavage was detected. (F) HCT116 cells were treated with STS, and GSDME cleavage was detected.
